# Atypical Mismatch Negativity in Response to Emotional Voices in People with Autism Spectrum Conditions

**DOI:** 10.1371/journal.pone.0102471

**Published:** 2014-07-18

**Authors:** Yang-Teng Fan, Yawei Cheng

**Affiliations:** 1 Institute of Neuroscience and Brain Research Center, National Yang-Ming University, Taipei, Taiwan; 2 Department of Rehabilitation, National Yang-Ming University Hospital, Yilan, Taiwan; 3 Department of Research and Education, Taipei City Hospital, Taipei, Taiwan; University of Jyväskylä, Finland

## Abstract

Autism Spectrum Conditions (ASC) are characterized by heterogeneous impairments of social reciprocity and sensory processing. Voices, similar to faces, convey socially relevant information. Whether voice processing is selectively impaired remains undetermined. This study involved recording mismatch negativity (MMN) while presenting emotionally spoken syllables *dada* and acoustically matched nonvocal sounds to 20 subjects with ASC and 20 healthy matched controls. The people with ASC exhibited no MMN response to emotional syllables and reduced MMN to nonvocal sounds, indicating general impairments of affective voice and acoustic discrimination. Weaker angry MMN amplitudes were associated with more autistic traits. Receiver operator characteristic analysis revealed that angry MMN amplitudes yielded a value of 0.88 (*p*<.001). The results suggest that people with ASC may process emotional voices in an atypical fashion already at the automatic stage. This processing abnormality can facilitate diagnosing ASC and enable social deficits in people with ASC to be predicted.

## Introduction

In Autism Spectrum Conditions (ASC), abnormalities in social skills usually coexist with atypical sensory processing and aberrant attention. Social deficits are characterized by difficulty in understanding others' mental status, including the recognition of emotional expressions through voices [1], [2]. Sensory dysfunction includes abnormalities in auditory processing, indicative of hyposensitivity or hypersensitivity to sounds [3], [4]. Aberrant attention typically shifts orientation from social to nonsocial stimuli [5]. To comprehensively understand the pathophysiology of autism, determining whether voice processing is selectively impaired in people diagnosed with ASC and whether this impairment is associated with sensory dysfunction and attention abnormalities is necessary.

Previous studies have suggested that ASC causes difficulty in encoding and representing the sensory features of physically complex stimuli [6]. Such a deficit causes people with autism to have a disadvantage when processing social information, because affective facial and vocal expressions are multifaceted. However, ASC does not cause certain types of complex auditory inputs, such as music, loudness, and pitch discrimination, to be misperceived [7], [8], [9]. Furthermore, people with ASC are considered to exhibit a fragmented mental representation and lack causative association because of slow voluntary attention shifting [10], [11]. A highly dynamic and interactive social realm should be highly susceptible to such impairments. However, studies on social-stimulus-specific deficits resulted from ASC have not distinguished sensory from attention processes nor have they evaluated the effects of physical stimulus complexity on their brain responses [5], [12].

Voice communication, a part of social interaction, is critical for survival [13], [14]. During the first few weeks following birth, infants can recognize the intonational characteristics of the languages spoken by their mothers [15], [16]. Typically developing infants can discriminate affective prosodies at 5 months of age [17] and react to affective components in vocal tones by 6 months of age [18]. However, young children with ASC do not show a preference for their mother's voice to other auditory stimuli [12], [19]. Adults with ASC exhibit difficulty in extracting mental state inferences from voices [1] and prosodies [20]. In a study of adults with ASC, the superior temporal sulcus, a voice-selective region, failed to activate in response to vocal sounds; however, the adults exhibited a normal activation pattern in response to nonvocal sounds [21]. Neurophysiological processing of emotional voices is atypical among people with ASC [22], [23].

Regarding superior temporal resolution, electroencephalographic event-related brain potentials (ERPs) enable the distinct stages of sensory and attentional processing to be examined. Mismatch negativity (MMN), which is elicited by perceptibly distinct sounds (deviants) in a sequence of repetitive sounds (standards), can be used to investigate the neural representation underlying automatic central auditory perception [24], [25]. Compared with standard stimuli, deviant stimuli evoke a more pronounced response at 100 to 250 ms and maximal amplitudes elicited over frontocentral regions [24]. The amplitude and latency of MMN indicate how effectively sound changes are discriminated from auditory background [26], [27], [28]. Recent studies have reported that MMN can be used as an index of the salience of emotional voice processing [29], [30], [31], [32].

Previous MMN findings regarding ASC are mixed [33]. When children with ASC were exposed to pitch changes in previous studies, the MMN responses were early peak latencies, [34], strong amplitudes [35], weak amplitudes [36], and no abnormality [11], [37], [38]. MMN was preserved when children with ASC attended to stimuli, but decreased in unattending conditions [39]. When presented with frequency deviants in streams of synthesized vowels, children with high-functioning ASC yielded MMN amplitudes compatible with those of controls [10]. MMN was preserved in response to nonspeech sounds, but diminished in response to speech syllables [19]. When elicited by one-word utterances, MMN in response to the neutral syllable as the standard, compared with the commanding, sad, and scornful deviants, was diminished in adults with Asperger's syndrome [23], whereas MMN elicited by commanding relative to tender voices in boys with Asperger's syndrome yielded the opposite result [22]. These discrepant findings may be related to population characteristics, stimulus features, and task designs. In particular, the corresponding acoustic parameters have not been controlled to a degree.

P3a that follows MMN is an ERP index of attentional orienting [40]. If deviants are perceptually salient, then an involuntary attention switch is generated to elicit P3a responses [10]. In a previous study, people with ASC exhibited P3a amplitudes similar to those of people with mental retardation and controls when inattentively listening to pure tones [34], [35]. Children with ASC exhibited P3a comparable to nonspeech sounds [41], but diminished responses to speech sounds [10], [11], [42]. Impaired attention orienting to speech-sound changes might affect social communication [10]. ASC cause speech-specific deficits in involuntary attention switching as well as normal orienting to nonspeech sounds.

To quantitatively control physical stimulus complexity, we presented meaningless emotionally spoken syllables, *dada*, and acoustically matched nonvocal sounds, representing the most and least complex stimuli, respectively, in a passive oddball paradigm, to people with ASC and matched controls. We hypothesized that people with ASC produce impaired MMN responses to emotional syllables and nonvocal sounds when general deficits in auditory processing are present. When the deficits are selective for voices, emotional syllables rather than nonvocal sounds diminish MMN responses among people with ASC. When involuntary attention orienting among people with ASC is speech-sound specific, P3a relevant to emotional syllables rather than nonvocal sounds would becomes atypical. In addition, to examine the relationship between electrophysiological responses and autistic traits, we conducted correlation analyses to determine the extent to which emotional MMN covaried with the Autism Spectrum Quotient (AQ) and receiver operating characteristic (ROC) analyses to evaluate the diagnostic utility of emotional MMN.

## Materials and Methods

### Participants

22 people with ASC and 21 matched controls participated in this study. Because of poor electroencephalogram (EEG) qualities, such as excessive eye movements and blink artifacts, 20 people with ASC and 20 controls were included in the data analysis. The participants with ASC, aged between 18 and 29 years (21.5±3.8 y, one female participant), were recruited from a community autism program. We reconfirmed the diagnosis of Asperger's syndrome and high-functioning autism by using Diagnostic and Statistical Manual of Mental Disorders (DSM)-IV diagnostic criteria as well as the Autism Diagnostic Interview-Revised (ADI-R) [43]. The participants in the age-, gender-, intelligence quotient (IQ)-, and handedness-matched control group, aged between 18 and 29 years (22.0±3.7 y, one female participant), were recruited from the local community and screened for major psychiatric illness by conducting structured interviews. The participants did not participate in any intervention or drug programs during the experimental period. Participants with a comorbid psychiatric or medical condition, history of head injury, or genetic disorder associated with autism were excluded. All of the participants exhibited normal peripheral hearing bilaterally (pure tone average thresholds <15 dB HL) at the time of testing. All of the participants or parents of the participants provided written informed consent for this study, which was approved by the Ethics Committee of Yang-Ming University Hospital and conducted in accordance with the Declaration of Helsinki.

### Auditory Stimuli

The stimulus materials were divided into two categories: emotional syllables and acoustically matched nonvocal sounds (Table S1 and Figure S1 in File S1). For emotional syllables, a female speaker from a performing arts school produced the meaningless syllables *dada* with three sets of emotional (neutral, angry, happy) prosodies. Within each set of emotional syllables, the speaker produced the syllables dada for more than ten times (see [29], [30], [31], [32] for validation). Emotional syllables were edited to become equally long (550 ms) and loud (min: 57 dB; max: 62 dB; mean 59 dB) using Sound Forge 9.0 and Cool Edit Pro 2.0. Each set was rated for emotionality on a 5-point Likert-scale. Two emotional syllables that were consistently identified as ‘extremely angry’ ad ‘extremely happy’ and one neutral syllables rated as the most emotionless were selected as the stimuli. The Likert-scale (mean ± SD) of angry, happy, and neutral syllables were 4.26±0.85, 4.04±0.91, and 2.47±0.87, respectively.

To create a set of control stimuli that retained acoustic correspondence, we synthesized nonvocal sounds by using Praat [44] and MATLAB (The MathWorks, Inc., Natick, MA, USA). The fundamental frequencies (f0) of emotional (angry, happy, neutral) syllables were extracted to produce the nonvocal sounds using a sine waveform and then multiplied by the syllable envelope. In this way, nonvocal sounds retained the temporal and spectral features of emotional syllables. All of the stimuli were controlled with respect to their length (550 ms) and loudness (min: 57 dB; max: 62 dB; mean 59 dB).

### Procedures

Before the EEG recordings were performed, each participant completed a self-administered questionnaire, the AQ, used for assessing autistic traits [45]. During the EEG recordings, participants were required to watch a silent movie with Chinese subtitles while task-irrelevant emotional syllables or nonvocal sounds in oddball sequences were presented. The passive oddball paradigm for emotional syllables involved employing happy and angry syllables as deviants and neutral syllables as standards. The corresponding nonvocal sounds were applied in the same paradigm but were presented as separate blocks. Each stimulus category comprised two blocks, the order of which was counterbalanced and randomized among the participants. Each block consisted of 600 trials, of which 80% were neutral syllables or tones, 10% were angry syllables or tones, and the remaining 10% were happy syllables or tones. The sequences of blocks and stimuli were quasirandomized such that the blocks of an identical stimulus category and the deviant stimuli were not presented successively. The stimulus-onset asynchrony was 1200 ms, including a stimulus length of 550 ms and an interstimulus interval of 650 ms.

### Electroencephalography Apparatus and Recordings

The EEG was continually recorded at 32 scalp sites. Please refer to Supplementary Materials (File S1) for details. The number of accepted standard and deviant trials between groups did not differ significantly irrespective of emotional syllables (ASC – Neutral: 750±149, Happy: 81±15, Angry: 83±11; Controls – Neutral: 746±112, Happy: 85±11, Angry: 83±13) or nonvocal sounds (745±189, 78±15, 76±17; 781±170, 78±11, 80±10). The paradigm was edited using MATLAB. Each event in the paradigm was associated with a digital code that was transmitted to the continual EEG, enabling offline segmentation and averages of selected EEG periods to be obtained for analysis. The ERPs were processed and analyzed using Neuroscan 4.3 (Compumedics Ltd., Australia).

MMN source distributions were qualitatively explored using current source density (CSD) mapping (http://psychophysiology.cpmc.columbia.edu/software/CSDtoolbox/index.html). The CSD method, as a measure of the strength of extracellular current generators underlying the recorded EEG potentials [46], computes the surface Laplacian over the surface potentials implying the dipole sources oriented normal to local skull [31], [47].

### Statistical Analysis

The MMN and P3a amplitudes were analyzed as an average within a 100-ms time window surrounding the peak latency at the electrode sites, Fz, Cz, and Pz according to previous knowledge [31], [32], [48]. The MMN peak was defined as the highest negativity in the subtraction between the deviant and standard sound ERPs, during a period of 150 to 250 ms after sound onset. Only the standards before the deviants were included in the analysis. The P3a peak was defined as the highest positivity during a period of 300 to 450 ms.

Statistical analyses were conducted, separately for each category (emotional syllables or nonvocal sounds), using a mixed ANOVA with deviant type (angry, happy), and electrode (Fz, Cz, or Pz) as the within-subject factors, and the group (ASC vs. control) as the between-subject factor with additional *a priori* group by deviant type ANOVA contrasts calculated within each electrode site [49]. The dependent variables were the mean amplitudes and peak latencies of the MMN and P3a components. Cohen's *d* was calculated to estimate the effect size (i.e., the standardized difference between means). Degrees of freedom were corrected using the Greenhouse-Geisser method. Bonferroni testing was conducted when preceded only by significant main effects.

To determine whether electrophysiological responses were associated with the severity of autistic traits, we conducted Pearson correlation analyses between MMN amplitudes and AQ scores. To examine the degree to which the MMN and P3a amplitudes could be used to differentiate between the participants with ASC and the controls, we conducted ROC analyses, which can identify optimal thresholds in diagnostic decision making.

## Results

### Demographics and Dispositional Measures

Table 1 lists the demographics and clinical variables of the participants. The ASC group, compared with the control group, scored higher on the AQ [*t*(34) = 5.08, *p*<.001, Cohen's *d* = 1.69] as well as on the subscales of social skill, attention switch, communication and imagination.

**Table 1:** Demographic and clinical variables of study participants.

	ASC (*N* = 20)		Controls (*N* = 20)		
	Mean	SD	Mean	SD	*p* value
**Age (yrs)**	21.5	3.8	22.0	3.7	.65
**IQ (WAIS)**	105	13.7	107	13.0	.61
**AQ**	29.4	5.6	21	4.8	<.001
Social skill	6.4	2.6	4.3	2.5	.013
Attention switch	6.9	1.6	5.7	1.5	.021
Attend to detail	5.8	1.9	5.1	2.0	.24
Communication	5.9	1.8	3.2	2.1	<.001
Imagination	4.4	1.5	2.5	1.8	.001

Abbreviations: IQ (WAIS), intelligence quotient assessed using the Wechsler Adult Intelligence Scale-Forth Edition (WAIS-IV) [68]; AQ, Autism Spectrum Quotient [45].

### Neurophysiological Measures

ERP amplitudes were subjected to an ANOVA in which the category (emotion syllables or nonvocal sounds), stimulus (happy, angry, or neutral), and electrode (Fz, Cz, or Pz) were repeated measure factors and the group (ASC vs. control) was the between-subject factor. The stimulus [*F* (2, 76) = 69.31, *p*<.001, *d* = 2.71] produced a main effect. The deviants elicited significantly stronger amplitudes than the standards did, regardless of whether they were emotional syllables or nonvocal sounds. In addition, significant interactions between the stimulus and group [*F* (2, 76) = 8.08, *p* = .001, *d* = 0.92], the category and stimulus [*F* (2, 76) = 6.93, *p* = .002, *d* = 0.85], the stimulus and electrode [*F* (4, 152) = 21.49, *p*<.001, *d* = 1.50], and the category, stimulus, and group [*F* (2, 76) = 3.25, *p* = .044, *d* = 0.58] were observed.

#### Emotional and Nonvocal Mismatch Negativity

**Figure 1:**
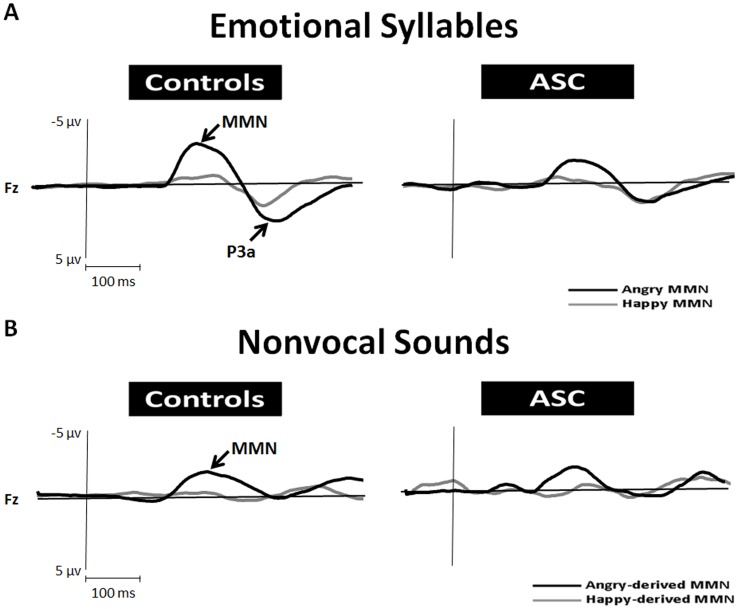
MMN amplitudes to emotional syllables and acoustically matched nonvocal sounds in people with ASC and controls at the electrode site Fz. MMN to angry deviants (black line) was significantly stronger in amplitude than MMN to happy deviants (gray line) in the controls (*p*<.001), whereas no differentiation was identified in people with ASC (*p* = .67). Nonvocal deviants that retained the acoustic features of emotional syllables were derived from angry (angry-derived) and happy (happy-derived) syllables. People with ASC exhibited weaker emotional-derived MMN than did the controls.

Automatic discrimination of emotional voices was examined using MMN, which was determined by subtracting the neutral ERP from angry and happy ERPs (Table S2 in File S1). According to the ANOVA model of emotional MMN amplitudes, the group [*F* (1, 38) = 6.69, *p* = .014, *d* = 0.84], deviant type [*F* (1, 38) = 21.03, *p*<.001, *d* = 1.49], and electrode site [*F* (2, 76) = 13.25, *p*<.001, *d* = 1.18] produced main effects. Participants with ASC exhibited weaker emotional MMN than the controls did. MMN in response to angry syllables (angry MMN) yielded stronger amplitudes than did MMN in response to happy syllables (happy MMN). Fz and Cz exhibited more negative deflections than did Pz. In addition, an interaction between the deviant type and the group [*F* (1, 38) = 15.13, *p*<.001, *d* = 1.26] was observed (Figure 1A). A post hoc analysis revealed that angry MMN were stronger than did happy MMN among the controls (*p*<.001), whereas no such difference was observed among the participants with ASC (*p* = .67).

To determine whether the MMN amplitude effects elicited by angry versus happy deviants between subject groups stemmed from differences in acoustic features, an additional MMN analysis was conducted by subtracting the neutral-derived ERP from the angry- and happy-derived ERPs. The ANOVA model indicated that the group [*F* (1, 38) = 4.38, *p* = .043, *d* = 0.68], deviant type [*F* (1, 38) = 52.22, *p*<.001, *d* = 2.35], and electrode site [*F* (2, 76) = 22.12, *p*<.001, *d* = 1.52] produced main effects. The people with ASC exhibited weaker MMN responses to nonvocal sounds than did the controls. Regardless of the group, MMN induced by angry-derived sounds (angry-derived MMN) was stronger than that elicited by happy-derived sounds (happy-derived MMN). Fz and Cz exhibited more negative deflections than did Pz. In addition, an interaction was observed between the deviant type and the electrode site [*F* (2, 76) = 11.08, *p*<.001, *d* = 1.08] (Figure 1B). A post hoc analysis indicated that the topographical distribution of angry-derived MMN yielded the most negative deflections at Fz and the least negative deflections at Pz. The happy-derived MMN exhibited no differential topography. Unlike emotional syllables, no interaction between the deviant type and the group was observed among nonvocal sounds (*p* = .65).

The ANOVA on the peak latency of MMN revealed that, regardless of the group, MMN in response to angry deviants peaked significantly later than did MMN in response to happy deviants [*F* (1, 38) = 13.38, *p* = .001, *d* = 1.19], but no such difference occurred in response to nonvocal deviants (*p* = .32). No significant MMN latency effect involving the group factor was observed in response to either emotional (*p* = .25) or nonvocal deviants (*p* = .55).

#### Emotional P3a

**Figure 2:**
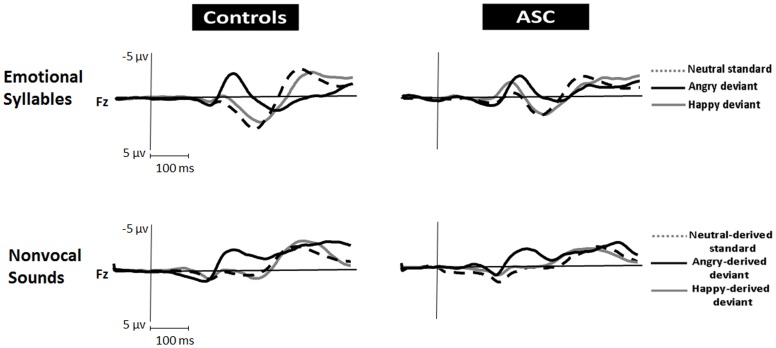
Grand average standard and deviant ERP waveforms for emotional syllables and acoustically matched nonvocal sounds in people with ASC and controls.

According to visual inspection, a P3a component was observed for only emotional syllables. The deviant type (angry or happy) and electrode site (Fz, Cz, or Pz) were the within-subject factors and the group (ASC vs. control) was the between-subject factor (Table S3 in File S1). The ANOVA revealed main effects in the deviant type [*F* (1, 38) = 13.49, *p* = .001, *d* = 1.19] and electrode site [*F* (2, 76) = 31.93, *p*<.001, *d* = 1.83]. P3a in response to angry syllables (angry P3a) yielded stronger amplitudes than did P3a in response to happy syllable (happy P3a). Fz exhibited the most positive deflections than did Cz and Pz. In addition, an interaction among the group, deviant type, and electrode site [*F* (2, 76) = 3.66, *p* = .029, *d* = 0.62]. A post hoc analysis revealed that angry P3a produced an interaction between the group and the electrode site [*F* (2, 76) = 3.89, *p* = .025, *d* = 0.64], but happy P3a did not (*p* = .96). People with ASC exhibited weaker angry P3a amplitudes than did the controls at Fz (*p* = .009). Figure 2 illustrates the ERP waveforms for standard and deviant responses.

#### Current Source Density Analyses

**Figure 3:**
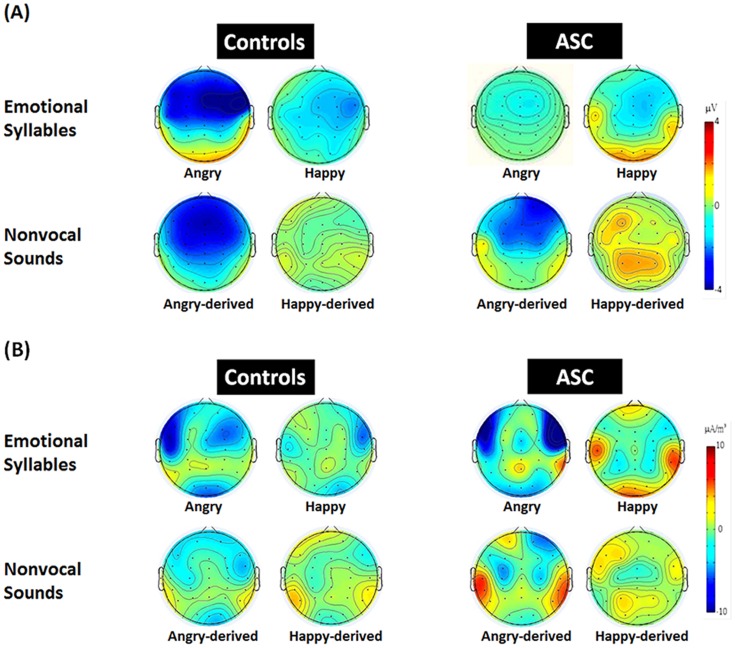
The MMN scalp potential distribution and the respective current source density (CSD) maps in people with ASC and controls. (A) A frontocentral minimum (or peak negativity) was similarly identified across the groups and categories. (B) The exploratory source distribution analyses on CSDs indicated that MMN received a major distribution from the bilateral auditory cortex. Additionally, for MMN to angry and angry-derived deviants, there was a trend toward a posterior temporal source in the ASC group.

The scalp topographies for absolute voltages of MMN for emotional syllables and nonvocal sounds in both groups were consistent with the MMN amplitudes results (Figure 3A). The exploratory source distribution analyses based on CSDs indicated that MMN received a major contribution from the auditory cortex (Figure 3B). In the ASC group, there was a trend toward an additional posterior temporal source.

#### Correlation Among Mismatch Negativity and Autistic Traits

**Figure 4:**
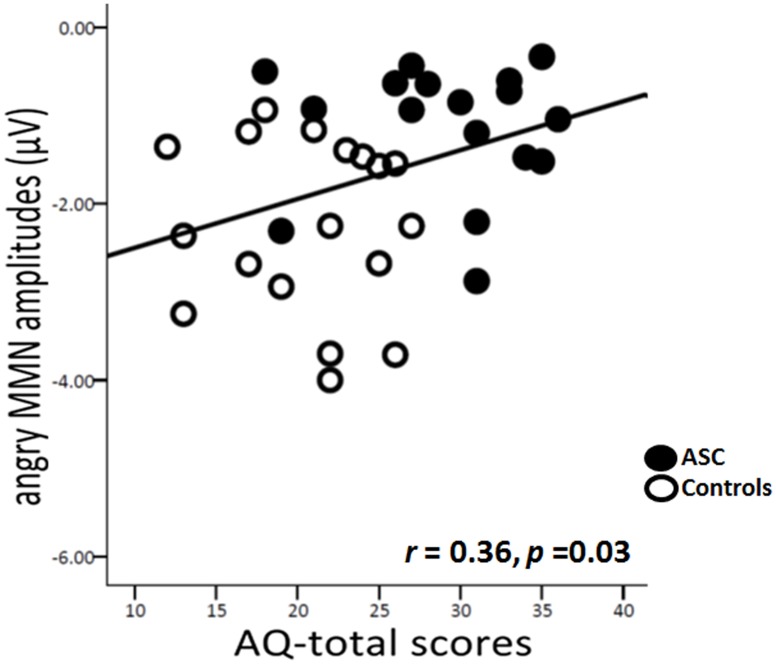
Correlation between angry MMN amplitudes and autistic traits.

When the two groups were combined, lower amplitudes of angry MMN at Fz were coupled with higher total scores on the AQ [*r* (36) = 0.36, *p* = .03, *d* = 0.77] (Figure 4). However, such a correlation was not observed in either the ASC group or the control group. MMN induced by nonvocal sounds did not exhibit any correlation. Also, there was no age-related correlation.

#### Relationship Between Sensitivity and Specificity for Angry Mismatch Negativity

**Figure 5:**
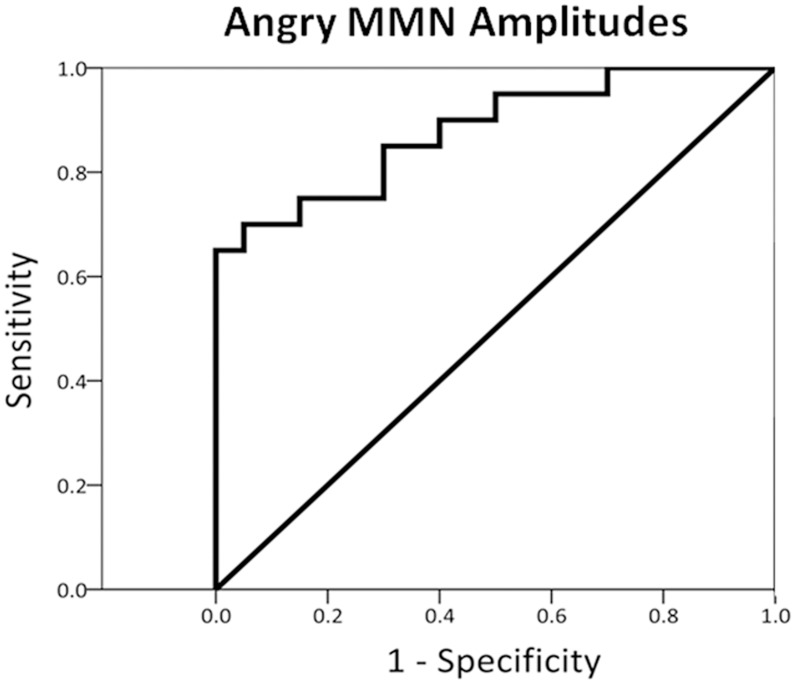
Receiver operator characteristic (ROC) analysis. The amplitude of angry MMN is suitable for predicting whether a person has a clinical diagnosis of ASC.

The area under the ROC curve (AUC) is indicative of the overall accuracy of the measurement, representing the probability that a randomly selected “true-positive” person scores higher according to the measure than a randomly selected “true-negative” person does. Separated ROC analyses for comparing the ASC participants with the controls were conducted for angry MMN, happy MMN, and angry-derived MMN, and happy-derived MMN. When determining optimal thresholds, we used Youden's index. This value corresponds with the point on the ROC curve farthest from the diagonal line. The diagonal line (sensitivity  = 0.5 and specificity  = 0.5) represents performance no better than chance. The ROC analysis of angry MMN yielded an AUC value of 0.88 (p<.001) (Figure 5). According to Youden's index, the most appropriate cutoff point for angry MMN amplitudes exhibiting a sensitivity of 95% and a specificity of 50% was −2.34 µV. By contrast, the AUC values of happy MMN, angry-derived MMN, and happy-derived MMN were not significant (p = .63; p = .14; p = .17).

## Discussion

This study investigated whether people with ASC exhibit selective deficits during emotional voice processing. The results indicated that people with ASC failed to exhibit differentiation between angry MMN and happy MMN. By contrast, in response to acoustically matched nonvocal sounds, people with ASC differentiated angry-derived MMN from happy-derived MMN to a low degree. P3a specific to emotional voices was reduced in people with ASC, indicating atypically involuntary attention switching. The significant correlation between the MMN amplitudes elicited by angry syllables and the total scores on the AQ indicated that angry MMN amplitudes were associated with autistic traits. ROC analyses revealed that angry MMN amplitudes yielded an AUC value of 0.88 (*p*<.001) for diagnosing ASC.

People with ASC failed to exhibit negativity bias in responses to emotional voices. In a previous study involving the same paradigm, we determined that negativity bias to affective voice emerges early in life [30]. Angry prosodies elicited a more negative-going ERP and stronger activation in the temporal voice area than did happy or neutral prosodies among infants [50]. Angry and fearful syllables evoked greater MMN than did happy or neutral syllables among adults and infants [30], [51]. A recent visual MMN study determined that an early difference occurred during 70 ms to 120 ms after stimulus onset for only fearful deviants under unattended conditions [52]. From an evolutionary perspective, threat-related emotion processing (e.g., anger and fear) is particularly strong and indicates independence of attention [53]. Negativity bias in affective processing occurs as early as evaluative categorization into valence classes does [54]. In this study, the stronger amplitudes observed in angry MMN compared with happy MMN among the controls were obscured among the people with ASC.

The human voice not only contains speech information but can also carry a speaker's identity and emotional state [55]. One MMN study determined that the MMN amplitudes were higher in response to intensity change in vocal sounds than in response to intensity change in corresponding nonvocal sounds. Although vocal intensity deviants may call for sensory and attentional resources regardless of whether they are loud or soft, comparable resources are recruited for nonvocal intensity deviants only when they are loud [56]. Thus, emotional syllables are considered to be more complex than nonvocal sounds and beyond low-level acoustic features [29], [30], [31], [32]. Because emotional MMN, instead of corresponding nonvocal sounds, exhibited a correlation with autistic traits and a positive predictive value for ASC, we speculated that low-level sensory deficits cannot be ascribed completely to social impairments in people with ASC.

In addition to lacking differentiation between angry and happy MMN, people with ASC exhibited reduced MMN in response to nonvocal sounds. The discrepancy between the results of this study and those of previous reports may be reflective of the heterogeneous characteristics of clinical participants, auditory stimuli, and task design [11], [34], [35]. For example, people with low-functioning autism might exhibit different MMN from those with high-functioning autism [35]. In one MMN study, basic acoustic features in the stimuli, specifically, emotional-neutral standards and emotional-laden deviants, were not controlled [23]. Furthermore, using one-word utterances or vowels as the auditory stimuli might cause variable familiarity or meaning, thus exerting potentially confounding effects on MMN responses [10], [22].

Involuntary attention orienting to emotional voices was atypical in people with ASC, as indicated by diminished P3a amplitudes to angry syllables. P3a is reflective of the involuntary capture of attention to salient environmental events [57]. In a previous study, vowels compared with corresponding nonvocal sounds, produced stronger P3a [10]. The attention-eliciting effect may be particularly pronounced when threat-related social information is involved [58]. We detected P3a for only emotional syllables, not for acoustically matched nonvocal sounds. Consistent with the results of previous studies [10], [59], [60], [61], our results indicated weaker P3a to emotional syllables among people with ASC compared with controls, suggesting that attention orienting in people with ASC is more selectively impaired to social stimuli than to physical stimuli.

In consistent with previous MMN studies [31], [62], our explorative CSD analyses suggested that the major contribution to deviance-standard difference responses comes from the bilateral auditory cortex. Furthermore, a slight trend toward to posterior enhancement observed in ASC for angry and angry-derived deviants could possibly reflect an additional posterior temporal source. The posterior lateral non-primary auditory cortex could be sensitive to emotion voices as indicated by functional neuroimaging [63]. However, given the known inaccuracies with EEG source localization, there CSD findings needs to be confirmed with more accurate source approaches.

ROC analyses revealed that the amplitudes of angry MMN yielded a sensitivity of 95% and a specificity of 50% for diagnosing ASC. Strong amplitudes of angry MMN were coupled with low total scores on the AQ when the ASC and control groups were combined. MMN changes can be reliably observed in people with autism [34], [64]. The AQ is a valuable instrument for rapidly determining where any given person is situated on the continuum from autism to normality [44]. AQ scores were determined to be associated with the ability to recognize mental state of others according to voices and eyes [65]. Thus, emotional MMM, particularly in response to angry syllables, is potentially useful as a neural marker for diagnosing autism.

Two limitations of this study must be acknowledged. First, regarding sample homogeneity, the generalizability of the results may be limited because people with low-functioning autism were not included. Second, stimuli that lack a quantitatively controlled function related to physical stimulus complexity, for instance, pure tones spectrally matching the fundamental frequency envelope of emotional syllables [29], [30], [31], [32], may limit the selectivity of emotional MMN. This may not be the optimal design, and future studies in which people with severe autism are recruited and a larger sample size and stimuli with greater acoustic correspondence are included are warranted.

## Conclusions

This study revealed that ASC involves general impairments in affective voice discrimination as well as low-level acoustic distinction. In addition to reduced amplitudes of MMN in response to acoustically matched nonvocal sounds, people with ASC failed to differentiate between angry and happy syllables. Weak amplitudes of angry MMN were coupled with severe autistic traits. The ROC analysis revealed that the amplitude of angry MMN is suitable for predicting whether a person has a clinical diagnosis of ASC. The ability to determine the likelihood of an infant developing autism by using simple neurobiological measures would constitute a critical scientific breakthrough [66]. Considering the advantages of clinical population assessment [67] and the presence of emotional mismatch response in the human neonatal brain [30], future studies must examine the ability of emotional MMN to facilitate the early diagnosis of infants at risk for ASC.

## Supporting Information

File S1 **Electroencephalography apparatus and recordings, Figure S1, and Tables S1–S3. Figure S1.** Acoustic properties of stimulus materials. **Table S1.** Physical and acoustic properties for the stimuli. **Table S2.** Mean amplitudes and peak latencies of MMN to emotional syllables and nonvocal sounds within a time window of 150 to 250 ms at predefined electrodes in each group (Mean ± SEM). **Table S3.** Mean amplitudes of P3a to emotional syllables within a time window of 300 to 450 ms at predefined electrodes in each group (Mean ± SEM).
(DOC)

## References

[1] Rutherford MD, Baron-Cohen S, Wheelwright S (2002) Reading the mind in the voice: a study with normal adults and adults with Asperger syndrome and high functioning autism. J Autism Dev Disord 32: 189–194. 12108620 10.1023/a:1015497629971

[2] Hobson RP, Ouston J, Lee A (1989) Naming emotion in faces and voices: abilities and disabilities in autism and mental retardation. Brit J Dev Psychol 7: 237–250.

[3] Mottron L (2011) Changing perceptions: The power of autism. Nature 479: 33–35. 22051659 10.1038/479033a

[4] Baron-Cohen S, Ashwin E, Ashwin C, Tavassoli T, Chakrabarti B (2009) Talent in autism: hyper-systemizing, hyper-attention to detail and sensory hypersensitivity. Philos Trans R Soc Lond B Biol Sci 364: 1377–1383. 19528020 10.1098/rstb.2008.0337 PMC2677592

[5] Dawson G, Meltzoff AN, Osterling J, Rinaldi J, Brown E (1998) Children with autism fail to orient to naturally occurring social stimuli. J Autism Dev Disord 28: 479–485. 9932234 10.1023/a:1026043926488

[6] Dawson G, Lewy A (1989) Autism: Nature, Diagnosis, and Treatment; Dawson G, editor. New York: Guilford.

[7] Mottron L, Peretz I, Menard E (2000) Local and global processing of music in high-functioning persons with autism: beyond central coherence? J Child Psychol Psychiatry 41: 1057–1065. 11099122

[8] Jarvinen-Pasley A, Wallace GL, Ramus F, Happe F, Heaton P (2008) Enhanced perceptual processing of speech in autism. Dev Sci 11: 109–121. 18171373 10.1111/j.1467-7687.2007.00644.x

[9] Khalfa S, Bruneau N, Roge B, Georgieff N, Veuillet E, et al (2004) Increased perception of loudness in autism. Hear Res 198: 87–92. 15617227 10.1016/j.heares.2004.07.006

[10] Čeponienė R, Lepistö T, Shestakova A, Vanhala R, Alku P, et al (2003) Speech-sound-selective auditory impairment in children with autism: they can perceive but do not attend. Proc Natl Acad Sci USA 100: 5567–5572. 12702776 10.1073/pnas.0835631100 PMC154385

[11] Lepistö T, Silokallio S, Nieminen-von Wendt T, Alku P, Naatanen R, et al (2006) Auditory perception and attention as reflected by the brain event-related potentials in children with Asperger syndrome. Clin Neurophysiol 117: 2161–2171. 16890012 10.1016/j.clinph.2006.06.709

[12] Klin A (1991) Young autistic children's listening preferences in regard to speech: a possible characterization of the symptom of social withdrawal. J Autism Dev Disord 21: 29–42. 1828067 10.1007/BF02206995

[13] Belin P, Grosbras MH (2010) Before speech: Cerebral voice processing in infants. Neuron 65: 733–735. 20346748 10.1016/j.neuron.2010.03.018

[14] Grossmann T, Friederici AD (2012) When during development do our brains get tuned to the human voice? Soc Neurosci 7: 369–372. 22017313 10.1080/17470919.2011.628758

[15] Mehler J, Jusczyk P, Lambertz G, Halsted N, Bertoncini J, et al (1988) A precursor of language acquisition in young infants. Cognition 29: 143–178. 3168420 10.1016/0010-0277(88)90035-2

[16] Vouloumanos A, Werker JF (2007) Listening to language at birth: evidence for a bias for speech in neonates. Dev Sci 10: 159–164. 17286838 10.1111/j.1467-7687.2007.00549.x

[17] Flom R, Bahrick LE (2007) The development of infant discrimination of affect in multimodal and unimodal stimulation: The role of intersensory redundancy. Dev Psychol 43: 238–252. 17201522 10.1037/0012-1649.43.1.238 PMC2704007

[18] Locke J (1993) The child's path to spoken language. Cambridge, MA: Harvard University Press.

[19] Kuhl PK, Coffey-Corina S, Padden D, Dawson G (2005) Links between social and linguistic processing of speech in preschool children with autism: behavioral and electrophysiological measures. Dev Sci 8: F1–F12. 15647058 10.1111/j.1467-7687.2004.00384.x

[20] Paul R, Augustyn A, Klin A, Volkmar FR (2005) Perception and production of prosody by speakers with autism spectrum disorders. J Autism Dev Disord 35: 205–220. 15909407 10.1007/s10803-004-1999-1

[21] Gervais H, Belin P, Boddaert N, Leboyer M, Coez A, et al (2004) Abnormal cortical voice processing in autism. Nat Neurosci 7: 801–802. 15258587 10.1038/nn1291

[22] Korpilahti P, Jansson-Verkasalo E, Mattila ML, Kuusikko S, Suominen K, et al (2007) Processing of affective speech prosody is impaired in Asperger syndrome. J Autism Dev Disord 37: 1539–1549. 17086440 10.1007/s10803-006-0271-2

[23] Kujala T, Lepistö T, Nieminen-von Wendt T, Näätänen P, Näätänen R (2005) Neurophysiological evidence for cortical discrimination impairment of prosody in Asperger syndrome. Neurosci Lett 383: 260–265. 15885908 10.1016/j.neulet.2005.04.048

[24] Näätänen R, Paavilainen P, Rinne T, Alho K (2007) The mismatch negativity (MMN) in basic research of central auditory processing: a review. Clin Neurophysiol 118: 2544–2590. 17931964 10.1016/j.clinph.2007.04.026

[25] Sussman ES, Chen S, Sussman-Fort J, Dinces E (2013) The Five Myths of MMN: Redefining How to Use MMN in Basic and Clinical Research. Brain Topogr. 10.1007/s10548-013-0326-6 PMC4000291 24158725

[26] Novitski N, Tervaniemi M, Huotilainen M, Näätänen R (2004) Frequency discrimination at different frequency levels as indexed by electrophysiological and behavioral measures. Brain Res Cogn Brain Res 20: 26–36. 15130586 10.1016/j.cogbrainres.2003.12.011

[27] Amenedo E, Escera C (2000) The accuracy of sound duration representation in the human brain determines the accuracy of behavioural perception. Eur J Neurosci 12: 2570–2574. 10947831 10.1046/j.1460-9568.2000.00114.x

[28] Kujala T, Kallio J, Tervaniemi M, Näätänen R (2001) The mismatch negativity as an index of temporal processing in audition. Clin Neurophysiol 112: 1712–1719. 11514254 10.1016/s1388-2457(01)00625-3

[29] Fan YT, Hsu YY, Cheng Y (2013) Sex matters: n-back modulates emotional mismatch negativity. Neuroreport 24: 457–463. 23660632 10.1097/WNR.0b013e32836169b9

[30] Cheng Y, Lee SY, Chen HY, Wang PY, Decety J (2012) Voice and emotion processing in the human neonatal brain. J Cogn Neurosci 24: 1411–1419. 22360593 10.1162/jocn_a_00214

[31] Hung AY, Ahveninen J, Cheng Y (2013) Atypical mismatch negativity to distressful voices associated with conduct disorder symptoms. J Child Psychol Psychiatry 54: 1016–1027. 23701279 10.1111/jcpp.12076 PMC3749266

[32] Hung AY, Cheng Y (2014) Sex differences in preattentive perception of emotional voices and acoustic attributes. Neuroreport 25: 464–469. 24488031 10.1097/WNR.0000000000000115

[33] O'Connor K (2012) Auditory processing in autism spectrum disorder: a review. Neurosci Biobehav Rev 36: 836–854. 22155284 10.1016/j.neubiorev.2011.11.008

[34] Gomot M, Giard MH, Adrien JL, Barthelemy C, Bruneau N (2002) Hypersensitivity to acoustic change in children with autism: electrophysiological evidence of left frontal cortex dysfunctioning. Psychophysiology 39: 577–584. 12236323 10.1017/S0048577202394058

[35] Ferri R, Elia M, Agarwal N, Lanuzza B, Musumeci SA, et al (2003) The mismatch negativity and the P3a components of the auditory event-related potentials in autistic low-functioning subjects. Clin Neurophysiol 114: 1671–1680. 12948796 10.1016/s1388-2457(03)00153-6

[36] Seri S, Cerquiglini A, Pisani F, Curatolo P (1999) Autism in tuberous sclerosis: evoked potential evidence for a deficit in auditory sensory processing. Clin Neurophysiol 110: 1825–1830. 10574297 10.1016/s1388-2457(99)00137-6

[37] Kemner C, Verbaten MN, Cuperus JM, Camfferman G, van Engeland H (1995) Auditory event-related brain potentials in autistic children and three different control groups. Biol Psychiatry 38: 150–165. 7578658 10.1016/0006-3223(94)00247-Z

[38] Jansson-Verkasalo E, Čeponienė R, Kielinen M, Suominen K, Jäntti V, et al (2003) Deficient auditory processing in children with Asperger Syndrome, as indexed by event-related potentials. Neurosci Lett 338: 197–200. 12581830 10.1016/s0304-3940(02)01405-2

[39] Dunn MA, Gomes H, Gravel J (2008) Mismatch negativity in children with autism and typical development. J Autism Dev Disord 38: 52–71. 17624605 10.1007/s10803-007-0359-3

[40] Escera C, Alho K, Schröger E, Winkler I (2000) Involuntary attention and distractibility as evaluated with event-related brain potentials. Audiol Neurootol 5: 151–166. 10859410 10.1159/000013877

[41] Lincoln AJ, Courchesne E, Harms L, Allen M (1993) Contextual probability evaluation in autistic, receptive developmental language disorder, and control children: event-related brain potential evidence. J Autism Dev Disord 23: 37–58. 8463201 10.1007/BF01066417

[42] Lepistö T, Kajander M, Vanhala R, Alku P, Huotilainen M, et al (2008) The perception of invariant speech features in children with autism. Biol Psychol 77: 25–31. 17919805 10.1016/j.biopsycho.2007.08.010

[43] Lord C, Rutter M, Le Couteur A (1994) Autism Diagnostic Interview-Revised: a revised version of a diagnostic interview for caregivers of individuals with possible pervasive developmental disorders. J Autism Dev Disord 24: 659–685. 7814313 10.1007/BF02172145

[44] Boersma P (2001) Praat, a system for doing phonetics by computer. Glot International 5: 341–345.

[45] Baron-Cohen S, Wheelwright S, Skinner R, Martin J, Clubley E (2001) The Autism-Spectrum Quotient (AQ): Evidence from Asperger Syndrome/High-Functioning Autism, Malesand Females, Scientists and Mathematicians. J Autism Dev Disord 31: 5–17. 11439754 10.1023/a:1005653411471

[46] Nicholson C (1973) Theoretical analysis of field potentials in anisotropic ensembles of neuronal elements. IEEE Trans Biomed Eng 20: 278–288. 4708762 10.1109/TBME.1973.324192

[47] Giard MH, Perrin F, Pernier J, Bouchet P (1990) Brain generators implicated in the processing of auditory stimulus deviance: a topographic event-related potential study. Psychophysiology 27: 627–640. 2100348 10.1111/j.1469-8986.1990.tb03184.x

[48] Duncan CC, Barry RJ, Connolly JF, Fischer C, Michie PT, et al (2009) Event-related potentials in clinical research: guidelines for eliciting, recording, and quantifying mismatch negativity, P300, and N400. Clin Neurophysiol 120: 1883–1908. 19796989 10.1016/j.clinph.2009.07.045

[49] Winer B, Brown D, Michels K (1991) Statistical Principles in Experimental Design.; McGrawhill, editor. New York.

[50] Grossmann T, Oberecker R, Koch SP, Friederici AD (2010) The developmental origins of voice processing in the human brain. Neuron 65: 852–858. 20346760 10.1016/j.neuron.2010.03.001 PMC2852650

[51] Schirmer A, Escoffier N, Zysset S, Koester D, Striano T, et al (2008) When vocal processing gets emotional: on the role of social orientation in relevance detection by the human amygdala. Neuroimage 40: 1402–1410. 18299209 10.1016/j.neuroimage.2008.01.018

[52] Stefanics G, Csukly G, Komlosi S, Czobor P, Czigler I (2012) Processing of unattended facial emotions: A visual mismatch negativity study. Neuroimage 59: 3042–3049. 22037000 10.1016/j.neuroimage.2011.10.041

[53] Vuilleumier P (2005) How brains beware: neural mechanisms of emotional attention. Trends Cogn Sci 9: 585–594. 16289871 10.1016/j.tics.2005.10.011

[54] Ito TA, Larsen JT, Smith NK, Cacioppo JT (1998) Negative information weighs more heavily on the brain: the negativity bias in evaluative categorizations. J Pers Soc Psychol 75: 887–900. 9825526 10.1037//0022-3514.75.4.887

[55] Belin P, Fecteau S, Bedard C (2004) Thinking the voice: neural correlates of voice perception. Trends Cogn Sci 8: 129–135. 15301753 10.1016/j.tics.2004.01.008

[56] Schirmer A, Simpson E, Escoffier N (2007) Listen up! Processing of intensity change differs for vocal and nonvocal sounds. Brain Res 1176: 103–112. 17900543 10.1016/j.brainres.2007.08.008

[57] Escera C, Alho K, Schroger E, Winkler I (2000) Involuntary attention and distractibility as evaluated with event-related brain potentials. Audiol Neurootol 5: 151–166. 10859410 10.1159/000013877

[58] Pratto F, John OP (1991) Automatic vigilance: the attention-grabbing power of negative social information. J Pers Soc Psychol 61: 380–391. 1941510 10.1037//0022-3514.61.3.380

[59] Lepistö T, Kujala T, Vanhala R, Alku P, Huotilainen M, et al (2005) The discrimination of and orienting to speech and non-speech sounds in children with autism. Brain Res 1066: 147–157. 16325159 10.1016/j.brainres.2005.10.052

[60] Maestro S, Muratori F, Cavallaro MC, Pei F, Stern D, et al (2002) Attentional skills during the first 6 months of age in autism spectrum disorder. J Am Acad Child Adolesc Psychiatry 41: 1239–1245. 12364846 10.1097/00004583-200210000-00014

[61] Dawson G, Toth K, Abbott R, Osterling J, Munson J, et al (2004) Early social attention impairments in autism: social orienting, joint attention, and attention to distress. Dev Psychol 40: 271–283. 14979766 10.1037/0012-1649.40.2.271

[62] Folstein JR, Van Petten C (2008) Influence of cognitive control and mismatch on the N2 component of the ERP: a review. Psychophysiology 45: 152–170. 17850238 10.1111/j.1469-8986.2007.00602.x PMC2365910

[63] Ethofer T, Bretscher J, Gschwind M, Kreifelts B, Wildgruber D, et al (2012) Emotional voice areas: anatomic location, functional properties, and structural connections revealed by combined fMRI/DTI. Cereb Cortex 22: 191–200. 21625012 10.1093/cercor/bhr113

[64] Roberts TP, Cannon KM, Tavabi K, Blaskey L, Khan SY, et al (2011) Auditory magnetic mismatch field latency: a biomarker for language impairment in autism. Biol Psychiatry 70: 263–269. 21392733 10.1016/j.biopsych.2011.01.015 PMC3134608

[65] Golan O, Baron-Cohen S, Hill JJ, Rutherford MD (2007) The ‘Reading the Mind in the Voice’ test-revised: a study of complex emotion recognition in adults with and without autism spectrum conditions. J Autism Dev Disord 37: 1096–1106. 17072749 10.1007/s10803-006-0252-5

[66] Griffin R, Westbury C (2011) Infant EEG activity as a biomarker for autism: a promising approach or a false promise? BMC Med 9: 61. 21599952 10.1186/1741-7015-9-61 PMC3117727

[67] Light GA, Braff DL (2005) Stability of mismatch negativity deficits and their relationship to functional impairments in chronic schizophrenia. Am J Psychiatry 162: 1741–1743. 16135637 10.1176/appi.ajp.162.9.1741

[68] Wechsler D (2008) Wechsler Adult Intelligence Scale-Fourth Edition. San Antonia, TX: Pearson.

