# Expression of Concern: Atypical Mismatch Negativity in Response to Emotional Voices in People with Autism Spectrum Conditions

**DOI:** 10.1371/journal.pone.0256413

**Published:** 2021-08-16

**Authors:** The PLOS ONE Editors

Following the publication of this article [1], concerns were raised regarding the unavailability of the auditory stimuli used in this study and the raw data underlying the results described in this study.

The authors clarified that they were unable to share the raw data underlying the reported results due to data-sharing restrictions implemented by the ethics approval obtained for this study, as well as the objections of some participants for their data to be shared with researchers for different research purposes. The authors provided a data set to the journal containing some of the processed data but did not provide the raw electroencephalogram (EEG) data. The processed data set is shared in the S1 File below. A board member assessed the processed data set provided by the authors and indicated that this dataset allows for replication of the statistical analysis only, and that the unprocessed raw EEG data are required to replicate the study findings.

In addition, the authors stated that the auditory stimuli used in this study cannot be shared publicly because these have been patented. Although the patent application was submitted prior to the article’s publication, this was not declared as a competing interest. The journal contacted the authors to request details of how others could obtain the stimuli, but the authors were unresponsive to this request. The board member consulted on this matter indicated that sharing of the auditory stimuli is critical for replicating the study’s findings.

The Data Availability Statement is updated to: “The raw data for this study cannot be made publicly available because the ethics approval and participant consent did not allow for data sharing with other researchers. Only the processed/aggregated data can be shared, this dataset is in S1 File.”

The Competing Interest statement is updated to: “At the time of the original article’s publication, Yawei Cheng was an Editorial Board member of *PLOS ONE*; this does not alter the author’s adherence to *PLOS ONE* Editorial policies and criteria. Furthermore, Yawei Cheng is listed as Inventor on the patent TW201408259 describing the emotional brain electric wave imaging method used in this study.”

The *PLOS ONE* Editors issue this Expression of Concern to notify readers of the discrepancy between the Data Availability Statement in the original publication indicating that data are fully available and the unavailability of the raw data. We also notify readers of the study’s reliance on patented auditory stimuli that have not been made publicly available. In light of these issues, the article does not comply in full with PLOS’ Materials Sharing policies.

## Supporting information

S1 File Processed data set. (SAV)
