# Supplementary material for: Atypical Mismatch Negativity in Response to Emotional Voices in People with Autism Spectrum Conditions
Source: PLoS One. 2014 Jul 18;9(7):e102471. doi: 10.1371/journal.pone.0102471 (PMC4103818; doi:10.1371/journal.pone.0102471)
Supplement: File S1 — Electroencephalography apparatus and recordings, Figure S1, and Tables S1–S3. Figure S1. Acoustic properties of stimulus materials. Table S1. Physical and acoustic properties for the stimuli. Table S2. Mean amplitudes and peak latencies of MMN to emotional syllables and nonvocal sounds within a time window of 150 to 250 ms at predefined electrodes in each group (Mean ± SEM). Table S3. Mean amplitudes of P3a to emotional syllables within a time window of 300 to 450 ms at predefined electrodes in each group (Mean ± SEM). (DOC) [file pone.0102471.s001.doc]

**Supplementary Materials**

**MATERIALS & METHODS**

**EEG Apparatus and Recordings**

The EEG experiment was conducted in a sound-attenuated, dimly light and electrically shielded room. Stimuli were presented binaurally via two loudspeakers placed approximately 30 cm distance on right and left sides of the subject’s head. The sound pressure level (SPL) peaks of different types of stimuli were equalized to eliminate the effect of the substantially greater energy of the angry stimuli. The mean background noise level was around 35 dB SPL. During EEG recording, the participants were required to watch a silent movie with Chinese subtitles while task-irrelevant emotional or nonvocal sounds in oddball sequences were presented.

The electroencephalogram (EEG) was continuously recorded from 32 scalp sites using electrodes mounted in an elastic cap and positioned according to the modified International 10−20 system, with the addition of two mastoid electrodes. The electrode at right mastoid (A2) was used as on-line reference. Eye blinks and vertical eye movements were monitored with electrodes located above and below left eye. The horizontal electro-oculogram (EOG) was recorded from electrodes placed 1.5 cm lateral to the left and right external canthi. A ground electrode was placed on the forehead. Electrode/skin impedance was kept < 5 kΩ. Channels were re-referenced off-line to the average of left and right mastoid recordings [(A1 + A2)/2]. Signals were sampled at 500 Hz, band-pass filtered (0.1−100 Hz), and epoched over an analysis time of 600 ms, including pre-stimulus 100 ms used for baseline correction. An automatic artifact rejection system excluded from the average all trials containing transients exceeding ±70μV at recording electrodes and exceeding ±100 μV at the horizontal EOG channel. Furthermore, the quality of ERP traces was ensured by careful visual inspection in every subject and trial and by applying appropriate digital, zero-phase shift band-pass filtered (0.1–50 Hz, 24 dB/octave). The first ten epochs of each sequence were omitted from the averaging in order to exclude unexpected large responses elicited by the initiation of the sequences.

**Figure s1: Acoustic properties of stimulus materials**.

**a.** Oscillogram and **b.** Spectrogram of auditory stimulus.


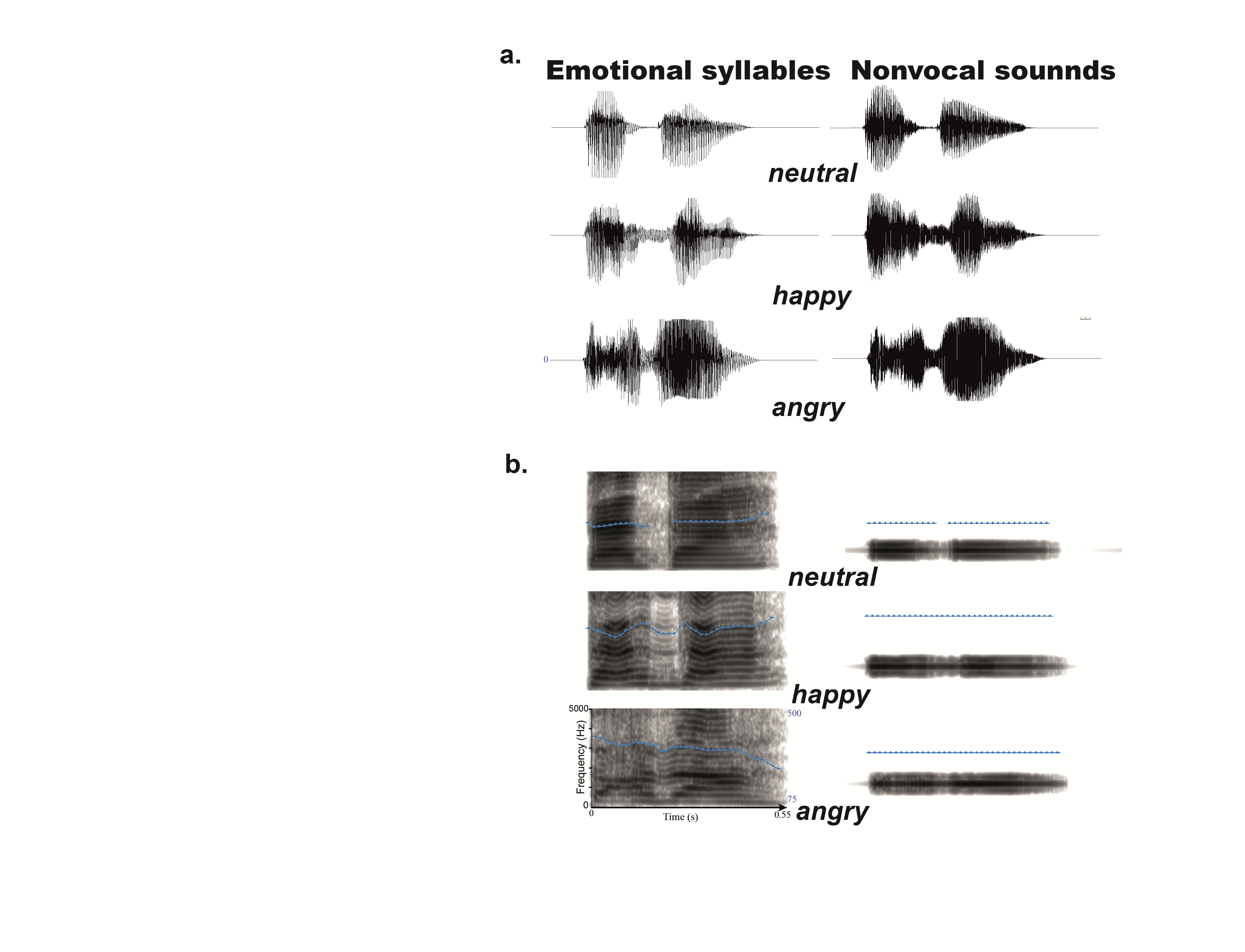


**Table s1.** Physical and acoustic properties for the stimuli

|  | Emotional syllables | | |  | Nonvocal sounds | | |
| --- | --- | --- | --- | --- | --- | --- | --- |
|  | intensity (dB) | duration (ms) | central frequency (Hz) |  | intensity (dB) | duration (ms) | central frequency (Hz) |
| Neutral | 57-62 | 550 | 1156 |  | 57-62 | 550 | 1156 |
| Happy | 57-62 | 550 | 1159 |  | 56-62 | 550 | 1159 |
| Angry | 57-62 | 550 | 1249 |  | 58-62 | 550 | 1249 |

**Table s2:** Mean amplitudes and peak latencies of MMN to emotional syllables and nonvocal sounds within a time window of 150 to 250 ms at predefined electrodes in each group (Mean ± SEM)

|  |  |  | **ASD** | |  | **Controls** | |
| --- | --- | --- | --- | --- | --- | --- | --- |
|  |  |  | Amplitude (µV) | Latency  (ms) |  | Amplitude (µV) | Latency  (ms) |
| **Emotional syllables** | **Angry** | Fz | -1.38±0.29 | 210.10±4.77 |  | -2.88±0.28 | 214.90±3.43 |
|  |  | Cz | -1.17±0.22 | 218.00±4.99 |  | -2.62±0.26 | 221.80±3.47 |
|  |  | Pz | -0.27±0.31 | 214.50±6.32 |  | -1.53±0.22 | 222.30±4.87 |
|  | **Happy** | Fz | -0.77±0.35 | 202.30±7.37 |  | -0.73±0.21 | 195.50±7.59 |
|  |  | Cz | -0.91±0.31 | 196.20±7.55 |  | -0.66±0.19 | 196.10±7.36 |
|  |  | Pz | -0.71±0.27 | 191.60±7.47 |  | -0.51±0.22 | 211.40±6.04 |
| **Nonvocal sounds** | **Angry-derived** | Fz | -1.38±0.36 | 207.70±5.81 |  | -1.85±0.26 | 208.40±4.29 |
|  |  | Cz | -0.95±0.28 | 209.50±5.41 |  | -1.53±0.23 | 214.00±4.83 |
|  |  | Pz | -0.11±0.28 | 201.10±6.36 |  | -0.94±0.19 | 203.80±5.58 |
|  | **Happy-derived** | Fz | 0.26±0.27 | 202.90±7.56 |  | -0.06±0.25 | 198.00±6.13 |
|  |  | Cz | 0.38±0.22 | 208.00±7.18 |  | 0.03±0.21 | 199.60±5.57 |
|  |  | Pz | 0.76±0.26 | 204.80±6.84 |  | 0.07±0.19 | 200.61±6.63 |

**Table s3.** Mean amplitudes of P3a to emotional syllables within a time window of 300 to 450 ms at predefined electrodes in each group (Mean ± SEM)

|  |  |  | **ASD** |  | **Controls** |
| --- | --- | --- | --- | --- | --- |
|  |  |  | Amplitude (µV) |  | Amplitude (µV) |
| **Emotional syllables** | **Angry** | Fz | 1.56±0.41 |  | 2.49±0.39 |
|  |  | Cz | 1.39±0.33 |  | 1.72±0.34 |
|  |  | Pz | 0.56±0.29 |  | 0.46±0.28 |
|  | **Happy** | Fz | 1.06±0.32 |  | 0.83±0.34 |
|  |  | Cz | 0.64±0.29 |  | 0.52±0.36 |
|  |  | Pz | 0.29±0.35 |  | 0.26±0.29 |
